# QuantiFERON-Cytomegalovirus Assay for Prediction of Cytomegalovirus Viremia in Kidney Transplant Recipients: Study From High Cytomegalovirus Seroprevalence Country

**DOI:** 10.3389/fcimb.2022.893232

**Published:** 2022-05-12

**Authors:** Kritsada Pongsakornkullachart, Methee Chayakulkeeree, Attapong Vongwiwatana, Wannee Kantakamalakul, Peenida Skulratanasak, Pakpoom Phoompoung

**Affiliations:** ^1^Department of Medicine, Faculty of Medicine Siriraj Hospital, Mahidol University, Bangkok, Thailand; ^2^Division of Infectious Diseases and Tropical Medicine, Department of Medicine, Faculty of Medicine Siriraj Hospital, Mahidol University, Bangkok, Thailand; ^3^Division of Nephrology, Department of Medicine, Faculty of Medicine Siriraj Hospital, Mahidol University, Bangkok, Thailand; ^4^Department of Microbiology, Faculty of Medicine Siriraj Hospital, Mahidol University, Bangkok, Thailand

**Keywords:** cytomegalovirus, QuantiFERON, transplant, immune, kidney transplant

## Abstract

**Background:**

Early studies showed the utility of pretransplant QuantiFERON-Cytomegalovirus (QF-CMV) assays for CMV-disease prediction post kidney transplant (KT). However, recent data are conflicting.

**Methods:**

This prospective cohort study enrolled adult patients undergoing KT between July 2017 and May 2019. Patients with antithymocyte globulin therapy or negative pretransplant CMV IgG were excluded. QF-CMV assays were performed on transplantation day and one month thereafter, and CMV viral loads were obtained 1, 3, and 6 months posttransplantation. The primary outcome was CMV viremia within 6 months. The QF-CMV assay–posttransplant CMV viremia association was analyzed.

**Results:**

Fifty-five patients were enrolled (male, 58.2%; mean (SD) age, 46.5 (10.2) years). Fifty-two (94.5%) received CMV-seropositive donor kidneys. Over 6 months, 29 patients developed CMV viremia (52.7%), with 14 (25.5%) having significant viremia requiring antiviral therapy. The CMV-viremia incidence of patients with nonreactive and reactive baseline QF-CMV assays did not differ significantly (55.3% and 47.1%; *p* = 0.573). Among patients with reactive pretransplant QF-CMV assays, there was a trend toward a lower incidence of CMV viremia for those who were persistently reactive at 1 month after KTs, although there was no statistically significant difference (50% vs 83%; *p* = 0.132).

**Conclusions:**

Our study could not support the use of single-timepoint pretransplant or 1-month posttransplant QF-CMV assays as a predictor for posttransplant CMV viremia in CMV seropositive KT recipients. Investigation of the association between dynamic QF-CMV-status changes and CMV-viremia incidence are needed.

## Introduction

Cytomegalovirus (CMV), a member of the human herpesvirus family, remains an important infectious complication in individuals with compromised immune functions, including solid organ transplant (SOT) recipients. Primary infection or reactivation of a latent infection usually occurs within six months of SOT (Fishman et al., 2007; Razonable and Humar, 2019). It has direct effects (CMV syndrome and tissue invasive diseases) and indirect effects (allograft dysfunction, rejection, and opportunistic infections) (Fishman et al., 2007; Razonable and Humar, 2019). CMV prevention is therefore an important measure to improve patient and graft survival.

The risk of CMV infection in SOT recipients depends on various factors, such as the organ transplanted and immunosuppressive therapy, but donor and recipient serological status are the most well-known risks. The 2 main strategies currently used to prevent CMV infection in SOT recipients are antiviral prophylaxis and preemptive management (Razonable and Humar, 2019). Traditionally, antiviral prophylaxis has been used as a preferable option in CMV-seronegative recipients receiving organs from CMV-seropositive donors (D+/R-), who have the highest risk of primary CMV infection (Humar et al., 2010). Either an antiviral prophylaxis or a preemptive approach is recommended in CMV-seropositive recipients (R+), who have an intermediate risk of CMV infection after transplantation (Razonable and Humar, 2019).

In Thailand, the kidney is the most common organ transplanted in clinical practice, with most kidney transplant (KT) recipients (99%) demonstrating CMV seropositivity (Watcharananan et al., 2012; Chiasakul et al., 2015). Previous studies reported that one-quarter of Thai patients developed CMV replication within 6 months of transplantation. Of those, antithymocyte globulin (ATG) therapy was strongly associated with an increased risk of CMV reactivation (Watcharananan et al., 2012; Chiasakul et al., 2015). Thus, better risk stratification is needed for seropositive KT recipients who do not receive ATG induction therapy in order to guide the choice of preventative measures.

Over the past decade, specific cell-mediated immunity has been studied to help stratify the risk of CMV infection in this population. The methods include commercially available tests—CMV enzyme-linked immunospot (ELISPOT) and QuantiFERON-CMV (QF-CMV) and nonstandardized laboratory methods, such as intracellular cytokine staining (ICS) (Yong et al., 2018). Of the commercial methods, ELISPOT is much more sensitive than the QF-CMV test (Yong et al., 2018). Nevertheless, it is costly and more labor intensive since a peripheral blood mononuclear cell isolation is needed. In addition, a lack of defined cutoff values for positivity is an issue of concern. By contrast, QF-CMV is easily performed in many diagnostic laboratories with a shorter turnaround time. Other studies have demonstrated the benefits of using a pretransplant QF-CMV to help stratify the risk of CMV infection in patients undergoing a kidney transplant (KT) (Lochmanova et al., 2010; Cantisan et al., 2013; Abate et al., 2013). Cantisan and colleagues investigated the use of pretransplant QF-CMV with 55 kidney and heart transplant recipients (Cantisan et al., 2013). The seropositive recipients who had a negative pretransplant QF-CMV assay were more likely to develop a CMV infection after their transplant than the nonreactive group (50% versus 13%, respectively). Nevertheless, recent published studies from Asia have provided conflicting results (Kwon et al., 2017; Lee et al., 2017). Hence, we performed this study to evaluate the benefits of using a QF-CMV assay to predict CMV infections in seropositive KT recipients.

## Materials and Methods

### Study Design and Study Population

We conducted a single-center, prospective cohort study at Siriraj Hospital, Mahidol University, Bangkok, Thailand. The study period was 23 months (July 1, 2017–May 31, 2019). All consecutive adult patients (aged 18 years or older) with CMV seropositivity admitted to the transplant unit for living-related-donor or deceased-donor KTs were eligible for enrollment. The exclusion criteria were patients undergoing a combined organ transplant, receiving valganciclovir or ganciclovir as a prophylaxis, or receiving ATG as induction therapy. The ethics committee at the study institute approved the research protocol.

### Procedures

Potential participants were assessed to confirm their eligibility prior to obtaining their written informed consent. Details of the baseline characteristics of the participants (such as their comorbidities, transplantation details, and donor history) and the use of immunosuppressive drugs were collected. Pre-transplant CMV immunoglobulin G (CMV IgG) was determined using chemiluminescent microparticle immunoassay (CMIA) method (Architect CMV IgG, Abbot, Illinois, USA). Five milliliters of plasma were obtained in a heparin tube for a QuantiFERON-CMV test (QIAGEN, Hilden, Germany) before the operation on the day of the KT (the “pre-KT QF-CMV”) and 1 month thereafter. Each sample was divided between three QF-CMV collecting tubes: a CMV-antigen tube, a nil-control tube, and a mitogen tube. The tubes were incubated, centrifuged, and assayed using the enzyme-linked immunosorbent method (ELISA), in accordance with the manufacturer’s instructions. The primary physicians were blinded to the results of the QF-CMV tests. A cutoff value (CMV-nil control) of 0.2 IU/mL was used for this study. A result of mitogen-nil < 0.5 IU/ml was considered an indeterminate result. Of note, patients with indeterminate results were included in the non-reactive group in the analysis.

Quantitative CMV nucleic acid testing (COBAS TaqMan CMV test; Roche, New Jersey, USA) performed in plasma was monitored at 1, 3 and 6 months after the KT. The lowest detected value was defined as 136 IU/ml. The signs and symptoms of a CMV infection were also monitored during the study period, and additional CMV testing was performed at the discretion of the primary physicians. The treatment of CMV diseases and preemptive management depended on the decision of the primary physician. Of note, since there has been no standard breakpoint of CMV viral load necessitating preemptive therapy, our single center observation showed a trend of disease progression among patients with CMV viral load exceeding 3 logs. As a result, we agreed to start treatment when the CMV viral load reached 1,000 IU/ml in our center.

### Definitions and Outcomes

CMV viremia was defined as any CMV viral load exceeding 136 IU/ml. Significant CMV viremia was any CMV viremia necessitating preemptive therapy (usually CMV viremia with level beyond 1,000 IU/ml, or rapid doubling time, or symptomatic CMV viremia). CMV syndrome and CMV diseases were classified according to the current guidelines of the American Society of Transplantation (Razonable and Humar, 2019). The primary outcome was the incidence of CMV viremia within 6 months of the KTs. The secondary outcomes were the incidence of significant CMV viremia and CMV diseases within 6 months of the KTs.

### Statistical Analysis

According to a previous study (Cantisan et al., 2013), the incidence of CMV viremia in nonreactive QF-CMV and reactive QF-CMV groups was 50% and 13%, respectively. To achieve power of 80% by using 2 proportion method, a sample size of 51 patients was estimated by calculation.

Categorical data, such as gender, are presented as frequency and percentage. Continuous data, for example, age, are reported as mean and standard deviation or median with range, depending on the data distribution. Inferential statistics were analyzed using the Chi-squared test or Fisher’s exact test. Quantitative statistics were analyzed with the independent-samples t test for normally distributed data, and the Mann–Whitney U test for data without a normal distribution. The data were recorded and analyzed using PASW Statistics for Windows (version 18.0; SPSS Inc., Chicago, Ill., USA), and *p*-values less than 0.05 were deemed statistically significant.

## Results

From July 2017 to May 2019, 67 adult patients underwent KTs at our institution. Sixty-five (97%) had CMV seropositivity at the time of transplantation. Ten patients were excluded from the study (four received ATG as an induction therapy; one received ganciclovir as a CMV prophylaxis; one died within one month of the transplant; one had a postponed KT; and three were excluded because of a shortage of QF-CMV test kits). The data related to the remaining 55 patients were analyzed.

The mean (SD) age of the patients was 46.5 (10.2) years, and the majority were men (32 patients; 58.2%). Diabetes was diagnosed in 7 patients (12.7%). The most common reason for the KT was end-stage renal disease of unknown etiology (32 patients; 58.2%), followed by end-stage glomerular disease (18 patients; 32.7%), autosomal dominant polycystic kidney disease (3 patients; 5.5%), and tubulointerstitial disease (1 patient; 1.8%). Two-thirds (69.1%) underwent a deceased-donor kidney transplantation (DDKT). Fifty-two patients (94.5%) received kidneys from CMV-seropositive donors. The mean (SD) age of the donors was 39 (13.4) years. All but one received basiliximab as an induction therapy. All patients received prednisolone, tacrolimus, or cyclosporin, plus mycophenolate mofetil for maintenance immunosuppression. Immunosuppressive agents were changed from calcineurin inhibitors to everolimus in only 2 patients. The mean dosage of prednisolone at 1^st^ and 6^th^ month posttransplant was 17 and 2.2 mg/day, respectively.

During the 6-month follow-up, CMV viremia occurred in 29 patients (52.7%); 14 of those patients (25.5%) had significant viremia, but none had CMV syndrome or CMV end-organ diseases. All but one developed CMV viremia within 3 months, with a mean (SD) onset of 65 (30) days. The median (range) CMV viral load for all CMV viremia and significant CMV viremia were 635 (157–81,363) and 1,800 (783–78,090) IU/ml, respectively. Infectious complications occurred in 29 patients (52.73%), of which urinary tract infections were the most common (17 patients; 58.62%), followed by BK polyomavirus infections (5 patients; 17.2%) and herpes simplex virus infections (3 patients; 10.3%). The infection incidence of the CMV-viremia and no-viremia groups did not differ significantly. A comparison of the baseline characteristics of the patients who did and did not develop CMV viremia is demonstrated in **Table 1**. The prednisolone and mycophenolate mofetil doses were slightly higher for the CMV viremia group; however, the tacrolimus trough level was lower for the CMV viremia group. A univariate analysis revealed that DDKT and increased donor age were the only factors associated with CMV viremia.

**Table 1:** Comparison of baseline characteristics of patients with and without CMV viremia.

Variables	CMV viremia (n = 29)	No CMV viremia (n = 26)	*P*-value
**Pretransplant data**			
Recipient age, years [mean (SD)]	47.31 (10.6)	45.7 (9.87)	0.478
Male sex, no (%)	18 (62.1%)	14 (53.8%)	0.537
Cause of ESRD, no (%)			
Glomerular disease	9 (31%)	9 (34.6%)	0.778
ADPKD	1 (3.4%)	2 (7.7%)	0.486
Tubulointerstitial disease	1 (3.4%)	0	0.347
Unknown	17 (58.6%)	15 (57.7%)	0.946
Comorbidities, no (%)			
Hypertension	15 (51.7%)	11 (42.3%)	0.485
Diabetes mellitus	5 (17.2%)	2 (7.7%)	0.426
Dyslipidemia	6 (20.7%)	2 (7.7%)	0.257
Chronic liver disease	3 (10.3%)	2 (7.7%)	1.000
Autoimmune disease	0	1 (3.8%)	0.473
**Transplant data**			
Retransplantation	0	2 (7.7%)	0.131
Donor type, deceased donor	25 (86.2%)	13 (50%)	0.004
Donor CMV seropositivity (D+)	26 (89.7%)	26 (100%)	0.238
Donor age, years [mean (SD)]	43.4 (15.1)	34.1 (9.1)	0.017
Induction therapy with basiliximab	28 (96.6%)	26 (100%)	0.347
Pretransplant leukocyte count, cells/mcl [mean (SD)]	5,892 (1,595)	6.503 (1,707)	0.176
Pretransplant lymphocyte count, cells/mcl [mean (SD)]	1,245 (486)	1,232 (453)	0.922
Cold ischemic time, hours [mean (SD)]	17 (4)	17 (3)	0.870
Delayed graft function, no (%)	11 (39.3%)	9 (33.3%)	0.646
Graft rejection	4 (13.8%)	0	0.113
Prednisolone dose at 1 month, mg [mean (SD)]	23.3 (2.9)	17.5 (10.6)	0.767
Mycophenolate dose at 1 month, mg [mean (SD))	960 (208)	405 (445)	0.823
Tacrolimus trough level at 1 month, ng/ml [mean (SD)]	7.3 (1.3)	8 (3)	0.045
QuantiFERON-CMV [median (range)]	1.52 (-0.11-10)	1.84 (-2.43-10)	0.785

CMV, cytomegalovirus; SD, standard deviation; ESRD, end stage renal disease; ADPKD, adult polycystic kidney disease.

### QF-CMV and CMV Viremia

Thirty-eight of the fifty-five enrolled patients (69.1%) had a QF-CMV reactive result on the day of their KT. At 1-month post-KT, 32/38 patients (84.2%) remained reactive, 5/38 had converted to a nonreactive status, and 1/38 had indeterminate result. Among 17 patients with a nonreactive pre-KT QF-CMV status, 13 patients were still nonreactive at 1-month after KTs, 4 patients had converted to indeterminate results.

When using the pretransplant QF-CMV results as a predictor of CMV infection, there was no difference between the incidence of CMV viremia for the patients with a nonreactive or indeterminate QF-CMV result, and that of the reactive group (55.3% vs 47.1%; *p* = 0.573). There was also no difference at 1 month after their KTs (51.5% vs 54.5%; *p* = 0.825). With a cutoff of 0.2 IU/ml, the sensitivity and specificity of the pre-KT QF-CMV assays were 27.6% and 65.4%, respectively. There were 3 patients receiving kidney from seronegative donor. All of them tested positive for baseline QF-CMV, and all developed CMV viremia but only one required CMV treatment. The association between QF-CMV and significant CMV viremia is detailed in **Table 2**.

**Table 2:** QF-CMV reactivity (> 0.2 IU/ml at pre-KT and 1 month) and incidence of CMV viremia.

Outcomes	Reactive QF-CMV Pre-KT QF-CMV (n = 38)	Non-reactive QF-CMV Pre-KT QF-CMV (n = 17)	*P*-value
CMV viremia within 1 month	7 (18.4%)	3 (17.6%)	1.000
CMV viremia within 3 months	20 (52.6%)	8 (47.1%)	0.702
CMV viremia within 6 months	21 (55.3%)	8 (47.1%)	0.573
Significant CMV viremia within 1 month	3 (7.9%)	2 (11.8%)	0.639
Significant CMV viremia within 3 months	9 (23.7%)	5 (29.4%)	0.742
Significant CMV viremia within 6 months	9 (23.7%)	5 (29.4%)	0.742
**Outcomes**	**Reactive QF-CMV** **1^st^ month QF-CMV** ** (n = 33)**	**Non-reactive/indeterminate** **1^st^ month QF-CMV** ** (n = 22)**	***P*-value**
CMV viremia within 1 month	7 (21.2%)	3 (13.6%)	0.723
CMV viremia within 3 months	16 (48.5%)	12 (54.5%)	0.660
CMV viremia within 6 months	17 (51.2%)	12 (54.5%)	0.825
Significant CMV viremia within 1 month	2 (60.6%)	3 (13.6%)	0.379
Significant CMV viremia within 3 months	6 (18.2%)	8 (36.4%)	0.129
Significant CMV viremia within 6 months	6 (18.2%)	8 (36.4%)	0.129

QF-CMV, Quantiferon CMV; CMV, cytomegalovirus; KT, kidney transplant.

In the case of patients who had a reactive pretransplant QF-CMV assay, the incidence of CMV viremia tended to be lower for those who remained QF-CMV reactive 1 month after their KTs (50% vs 83%; *p* = 0.132). Sixty percent of patients (3/5) who had an indeterminate result 1-month posttransplant developed CMV viremia, while 52% of the patients who had a reactive or nonreactive result developed the same outcome (*p* = 1.000).

Of note,13 patients showed a strong QF-CMV response (CMV-nil control > 10). A further analysis using QF-CMV > 10 IU/ml as the defined cutoff value was performed. Although patients with a reactive pretransplant QF-CMV (2/13; 15.4%) were less likely to develop significant CMV viremia than those in the nonreactive group (13/42; 31%), the difference was not statistically significant. We also noticed that two patients with a pre-KT reactive QF-CMV > 10 IU/ml who had significant CMV viremia demonstrated a relatively lower CMV viral load at CMV diagnosis when compared with those who had QF-CMV < 10 IU/ml (1,016 and 3,256 IU/ml, respectively).

## Discussion

Our study could not demonstrate the value of pretransplant QF-CMV status as a reliable predictor for CMV infections in KT recipients with CMV seropositivity who were classified as intermediate-risk group for CMV infection. Although data from earlier studies support the benefits of obtaining a pretransplant QF-CMV in the high-risk population, especially for patients with a CMV serological mismatch (D+R-) (Kumar et al., 2009; Manuel et al., 2013), the benefits are still unclear for the intermediate-risk group. Our results support a previous study performed by Lee and colleagues (Lee et al., 2017), who compared the efficacies of QF-CMV and ELISPOT assays in KT recipients. They reported a lack of association between pretransplant QF-CMV reactivity and the incidence of CMV viremia after KTs in the intermediate-risk population. In contrast, they found that a CMV-ELISPOT assay performed 1-month posttransplant provided more reactive result and potentially be used as a predictor for CMV replication in this group. This finding was also confirmed by a recent meta-analysis undertaken by Ruan et al. in 2019 (Ruan et al., 2019), who reported a lower sensitivity with QF-CMV (38%) than CMV-ELISPOT (73%–84%).

There are 3 possible explanations for the superior performance of ELISPOT relative to QF-CMV. Firstly, even though both tests use the same principle of IFN-gamma release assay, the QF-CMV assay relies on the detection of interferon-gamma (IFN-γ) in whole blood, whereas ELISPOT directly detects the virus-specific T lymphocytes that produce IFN-γ, which demonstrates functioning T-lymphocytes *in vivo*. This hypothesis correlates with intracellular cytokine staining which also has the additional ability to detect multiple cytokines and cell-surface markers (Fernandez-Ruiz et al., 2019; Rogers et al., 2020). Secondly, QF-CMV can only detect the cytotoxic T-cell (CD8) response, whereas ELISPOT can detect the response from both the helper (CD4) and CD8. (Waldman and Knight, 1996; Snyder et al., 2016). Lastly, QF-CMV requires the *ex vivo* stimulation of CD8 T-cells with human leukocyte antigen (HLA)-restricted CMV peptides, while ELISPOT is usually performed with peptide library spanning the whole antigens pp65 and IE-1. (Giulieri and Manuel, 2011). IE-1 could be crucial in CMV seropositive patients (Lopez-Oliva et al., 2014) while only 4 peptides presented by specific HLA alleles are included in QF-CMV (Walker et al., 2007). Lack of these HLA alleles can also cause false-negative QF-CMV tests.

The data from another study showed that patients with indeterminate QF-CMV results had an increased incidence of CMV disease or serious infectious complications (Tarasewicz et al., 2016). These findings were explained by patients’ high net state of immunosuppression since patients with an indeterminate result could not stimulate the mitogen. However, our study did not support this finding. There were indeterminate results for the QF-CMV assay (1 month after the KTs) in only 9% of cases, which was much lower than previously reported in the literature (Kumar et al., 2009). This finding comes as no surprise since our patients were not highly immunosuppressive, having relatively low FK levels (Lochmanova et al., 2010; Abate et al., 2013; Kwon et al., 2017). This might explain the negative association of the indeterminate result.

The appropriate timeline and cutoff value for QF-CMV are not yet clear. Some studies have reported a better utility with cell-mediated immune monitoring 1 month after a KT, which is the period of maximal immunosuppressant levels (Lee et al., 2017). However, our study could not demonstrate any benefit at this timepoint. Regarding the appropriate cutoff value, we hypothesized that 0.2 IU/ml might be suitable only for CMV-seronegative patients; a higher cutoff value might be required for seropositive recipients exposed to a CMV infection before their KT. Some studies have employed a higher cutoff value to predict CMV disease in transplant recipients (Gliga et al., 2018; Krawczyk et al., 2018). For example, data from bone marrow transplant recipients indicated that there was a need for a higher QF-CMV cutoff value (> 8.9 IU/ml) to stratify the risk of CMV disease in that population (Tarasewicz et al., 2016). Further analysis using QF-CMV > 10 IU/ml as the cutoff value revealed that patients with a reactive pretransplant QF-CMV (15.4%) were less likely to develop significant CMV viremia than those in the nonreactive group (31%). However, the difference did not reach statistical significance. Additional investigation with a larger sample size is required to determine a better cutoff value for intermediate-risk KT recipients.

Interestingly, although a single-timepoint, pretransplant, QF-CMV assay or a 1-month, post-KT, QF-CMV assay might not predict CMV viremia in KT recipients, further analysis found that the incidence of CMV viremia tended to be lower in those patients remaining reactive 1 month after their KTs. These findings suggest that dynamic changes in QF-CMV assays could be more helpful for predicting the incidence of CMV viremia in seropositive KT recipients. As a result, multiple time points of QF-CMV and larger sample sizes are needed to characterize the utility of dynamic changes in the QF-CMV levels to predict CMV viremia after a transplant.

The strength of our study is the homogeneity of the study population in terms of the recipients’ serostatuses and degrees of immunosuppression. Our main limitation is the small sample size. Moreover, the relatively low frequency of CMV QNAT monitoring in this study may have resulted in the missing of some episodes of CMV viremia with spontaneous viral clearance, thereby causing an outcome measurement bias.

## Conclusions

In conclusion, our study could not confirm that single-timepoint pretransplant QF-CMV assay or a 1-month post-KT QF-CMV assay are a useful marker for predicting posttransplant CMV viremia in seropositive KT patients. Further studies with larger sample size are needed to confirm these findings. Multiple time points to evaluate the changes of QF-CMV level with a defined cutoff value may be useful to predict CMV viremia.

## Data Availability Statement

The original contributions presented in the study are included in the article/supplementary material. Further inquiries can be directed to the corresponding author.

## Ethics Statement

The studies involving human participants were reviewed and approved by Siriraj Institutional Review Board. The patients/participants provided their written informed consent to participate in this study.

## Author Contributions

KP: investigation, formal analysis, writing-original draft; MC: writing-review and editing, supervision; AV: resources, writing-review and editing; WK: resources, writing-review and editing; PS: resources, writing-review and editing; PP: conceptualization, methodology, writing-review and editing, visualization. All authors contributed to the article and approved the submitted version.

## Funding

This study was supported by a research development grant from Siriraj Hospital (Grant number R016032034). Although the test kits were provided by the Qiagen company, it played no role in the study design, data collection, study analysis, or manuscript writing.

## Conflict of Interest

The authors declare that the research was conducted in the absence of any commercial or financial relationships that could be construed as a potential conflict of interest.

## Publisher’s Note

All claims expressed in this article are solely those of the authors and do not necessarily represent those of their affiliated organizations, or those of the publisher, the editors and the reviewers. Any product that may be evaluated in this article, or claim that may be made by its manufacturer, is not guaranteed or endorsed by the publisher.
